# Prognostic value of *BCL2* and *TP53* genetic alterations for diffuse large B-cell lymphoma patients treated with R-CHOP

**DOI:** 10.20892/j.issn.2095-3941.2021.0193

**Published:** 2021-11-23

**Authors:** Yan Qin, Haizhu Chen, Peng Liu, Changgong Zhang, Jianliang Yang, Lin Gui, Xiaohui He, Liqiang Zhou, Shengyu Zhou, Shiyu Jiang, Hongxin Jiang, Yuankai Shi

**Affiliations:** 1 Department of Medical Oncology, National Cancer Center/National Clinical Research Center for Cancer/Cancer Hospital, Chinese Academy of Medical Sciences & Peking Union Medical College, Beijing Key Laboratory of Clinical Study on Anticancer Molecular Targeted Drugs, Beijing 100021, China; 2 Department of Medical Oncology, Suzhou Municipal Hospital, Affiliated Suzhou Hospital of Nanjing Medical University, Suzhou 215001, China

**Keywords:** BCL2, TP53, genetic alterations, diffuse large B-cell lymphoma, prognosis

## Abstract

**Objective:**

Limited data about the prognostic significance of *BCL2* mutations and *BCL2* copy number variations in diffuse large B-cell lymphoma (DLBCL) are available. This study aimed to comprehensively describe *BCL2* genetic alterations in DLBCL patients, and examine correlation of *BCL2*, *TP53* and other genetic alterations with outcomes in patients treated with R-CHOP.

**Methods:**

Probe capture-based high-resolution sequencing was performed on 191 patients diagnosed with *de novo* DLBCL. MYC, BCL2, and BCL6 protein expressions were detected by immunohistochemistry.

**Results:**

The presence of *BCL2* alterations significantly correlated with poor progression-free survival (PFS) (5-year PFS: 13.7% *vs.* 40.8%; *P* = 0.003) and overall survival (OS) (5-year OS: 34.0% *vs.* 70.9%; *P* = 0.036). Importantly, patients who harbored *BCL2* gain/amplifications (*BCL2*^GA/AMP^) also had a remarkably inferior 5-year PFS (11.1% *vs.* 38.3%; *P* < 0.001) and OS (22.1% *vs.* 69.6%; *P* = 0.009). In contrast, neither *BCL2* mutations nor *BCL2* translocations were significantly prognostic for survival. Multivariable analyses showed that the presence of *BCL2* alterations, especially *BCL2*^GA/AMP^, *TP53* mutations, and International Prognostic Index (IPI) were significantly associated with inferior PFS and OS. Novel prognostic models for OS were constructed based on 3 risk factors, including *BCL2* alterations (Model 1) or *BCL2*^GA/AMP^ (Model 2), *TP53* mutations, and IPI, to stratify patients into 4 risk groups with different survival outcomes.

**Conclusions:**

This study showed that DLBCL patients treated with R-CHOP, *BCL2* alterations, especially *BCL2*^GA/AMP^ and *TP53* mutations were significantly associated with inferior outcomes, which were independent of the IPI. The novel prognostic models we proposed predicted outcomes for DLBCL patients treated with R-CHOP, but further validation of the prognostic models is still warranted.

## Introduction

Diffuse large B-cell lymphoma (DLBCL), the most common subtype of lymphoma in adults, can be successfully treated by standard immunochemotherapy in 50%–60% of patients^1,2^. To predict outcomes before treatment, the International Prognostic Index (IPI) model for aggressive non-Hodgkin lymphoma based on chemotherapy was developed in 1993^3^. Using immunochemotherapy, the addition of rituximab to cyclophosphamide, doxorubicin, vincristine, and prednisone (R-CHOP) has led to an improvement in survival^4,5^. In the rituximab treatment era, redistribution of the IPI factors into a revised IPI (R-IPI) provides a more clinically meaningful prognostic prediction^6^. Although IPI and R-IPI are useful tools for risk stratification of patients with DLBCL, all the risk factors are clinical features, which does not describe the biological spectrum.

The molecular heterogeneity of DLBCL is considered to be related to different therapeutic outcomes of chemotherapy and immunochemotherapy^7–9^. The initial progress using a genetic description to predict clinical outcomes involved gene expression profiling, which distinguished 2 subtypes, including activated B-cell like (ABC) and germinal center B-cell like (GCB) in the cell-of-origin (COO) classification of DLBCL^8,9^. However, the COO distinction does not fully account for the heterogeneous responses and outcomes following R-CHOP. Studies using next-generation sequencing have characterized the mutational landscape and identified the genetic drivers of DLBCL^7,10,11^. Several gene mutations including *MYD88*^L265P^ and *CD79B* mutations, *NOTCH1* mutations, and *TP53* mutations are independent risk factors related to poor prognosis in DLBCL patients^7,10–15^. Additionally, recent studies have emphasized the prognostic role of the tumor microenvironment (TME) in DLBCL, and several biomarkers related to the TME have thus been identified^16–21^.

BCL2, mainly involving translocations and protein expression, has been extensively investigated as a prognostic biomarker ins DLBCL, but with controversial findings^22–25^. There is general consensus that patients with concurrent *MYC* and *BCL2* or *BCL6* rearrangements, referred to as double hit lymphoma (DHL), have an extremely aggressive clinical course and poor prognosis. However, DHL is relatively rare, representing only 4%–8% of DLBCL patients^26,27^. In contrast, the predictive value of other *BCL2* genetic alterations has been less studied. In particular, limited information about the prognostic significance of *BCL2* mutations and *BCL2* copy number variations (CNV) are available, and no consistent results have yet been reported^26,28,29^.

In this study, we performed capture-based targeted sequencing on 191 Chinese DLBCL patients, to comprehensively describe *BCL2* genetic alterations. We also determined the correlations of clinicopathological features, *BCL2*, *TP53*, and other genetic alterations with outcomes in patients treated with R-CHOP.

## Materials and methods

### Study population

A total of 205 patients diagnosed with *de novo* DLBCL at the National Cancer Center/National Clinical Research Center for Cancer/Cancer Hospital, Chinese Academy of Medical Sciences & Peking Union Medical College, and Suzhou Municipal Hospital, from January 2004 to January 2020, were selected for this study. Histological diagnoses were established according to the World Health Organization classification of tumors of hematopoietic and lymphoid tissues in 2008^30^. The inclusion criteria were as follows: 1) patients with histologically confirmed DLBCL; 2) patients who had adequate tissue for DNA extraction and who provided informed consent; 3) patients initially treated with a curative intent; and 4) patients without human immunodeficiency virus infection. Individuals diagnosed with primary central nervous system DLBCL, with incomplete survival data, with a history of an indolent lymphoma, or other primary malignancies were excluded, resulting in a total of 191 patients included in this study. Only 164 patients who received R-CHOP or R-CHOP-like regimens [including R-mini CHOP, R-CDOP (rituximab, cyclophosphamide, pegylated liposomal doxorubicin, vincristine, and prednisone), R-CHO (rituximab, cyclophosphamide, doxorubicin, and vincristine), and R-CHOPE (rituximab, cyclophosphamide, doxorubicin, vincristine, and prednisone etoposide)] were included for survival and prognostic analyses. The selection process is described in **Supplementary Figure S1**.

Baseline clinical characteristics and follow-up information were collected, including age, gender, Eastern Cooperative Oncology Group (ECOG) performance status (PS), Ann Arbor stage, primary sites, number of extranodal involvement sites, IPI scores, lactate dehydrogenase (LDH) levels, response to treatment, and survival data. Ann Arbor stage was categorized into 2 stage groups involving the limited stage (I–II) and advanced stage (III–IV). IPI was grouped into 2 risk groups involving the low/low-intermediate group (IPI score: 0–2) and the high-intermediate/high group (IPI score: 3–5). This study was performed in accordance with the Declaration of Helsinki, and approved by the Institutional Review Board of the National Cancer Center/National Clinical Research Center for Cancer/Cancer Hospital, Chinese Academy of Medical Sciences & Peking Union Medical College (No. NCC2018JJJ-004). Written informed consent was obtained from all patients.

### Capture-based targeted DNA sequencing

Archived formalin-fixed paraffin-embedded (FFPE) tissue samples were obtained from enrolled patients. Matched peripheral blood was also collected as the germline control. Library construction was performed based on genomic DNA extracted from FFPE using the QIAamp DNA FFPE Tissue Kit (Qiagen, Hilden, Germany), and from peripheral blood using the QIAamp DNA Blood Mini Kit (Qiagen). The concentration of DNA was assessed using a Qubit fluorometer and Qubit dsDNA HS (High Sensitivity) Assay Kit (Invitrogen, Carlsbad, CA, USA). The Agilent 2100 Bio Analyzer and the DNA HS Kit (Agilent Technologies, Santa Clara, CA, USA) were used to measure the distributions of plasma DNA. DNA was fragmented into 200–250 bp sizes using a Covaris S2 Ultrasonicator (Covaris, Woburn, MA, USA). Hybridization with capture probe baits, hybrid selection with magnetic beads, and the polymerase chain reaction amplification were subsequently conducted. Two capture probes covering genes that were commonly altered in human lymphoma and hematological malignancies were selected, with 1 covering 112 genes and another consisting of 413 genes. A total of 101 genes overlapped between the 2 panels^14^. Sample sequencing was performed on a Next Seq500 Sequencer (Illumina, Hayward, CA, USA) with pair-end reads at Burning Rock Biotech (Guangzhou, China) or Geneplus-Beijing (Beijing, China). The detailed sequencing procedure was performed as described previously^14,15,31^.

### Sequencing data analysis

After removal of terminal adapter sequences and low quality data, sequencing data were mapped to the reference human genome (hg19) and assigned with a Burrows-Wheeler assigner 0.7.10 (Broad Institute, Cambridge, MA, USA)^32^. GATK 3.2 and MuTect (both from Broad Institute), and VarScan (Genome Institute, Washington University, St. Louis, MO, USA) were used to perform local alignment optimization, variant calling, and annotation^33,34^. The VarScan filter pipeline was used to filter-out loci with a depth < 100. Single nucleotide variants were determined using MuTect (version 1.1.4) and NChot^35,36^. The average sequencing depth for all targeted regions was 1,402×. Selected exons of several genes of interest overlapping in the 2 panels, including *BCL2*, *MYC*, *BCL6*, *TP53*, *NOTCH1*, *MYD88*, and *CD79B*, were analyzed. In accordance with the Exome Aggregation Consortium, 1,000 Genomes Project, ESP6500SI-V2, and dbSNP databases, single nucleotide polymorphisms, which were defined as variants with a frequency > 0.1%, were excluded from further analysis. The remaining variants were annotated with ANNOVAR as well as SnpEff v.3.6 software.

CNV analysis was performed based upon the depth of coverage data of capture intervals. Coverage data were corrected against sequencing bias resulting from GC content and probe design. Based on the average coverage of all capture regions, the coverage of different samples was normalized to comparable scales. Copy number was computed based on the ratio between the depth of coverage in tumor samples and the average coverage of an adequate number (*n* > 50) of samples without CNVs as referenced per capture interval. CNV was defined when the coverage data of the gene region was quantitatively and significantly different from the reference control. CNV detection with a threshold value ≤ 1.5 was defined as loss, and a threshold ≥ 2.64 was referred to as gain or amplification. The copy number gains or amplifications of genes, including the *BCL2*, *MYC*, and *BCL6* genes, were grouped into a single group, and designated as *BCL2*^GA/AMP^, *MYC*^GA/AMP^ or *BCL6*^GA/AMP^. Analysis of DNA translocation was performed using Tophat 2 (Center for Computational Biology, Johns Hopkins University, Baltimore, MD, USA and the Genome Sciences Department, University of Washington, Seattle, WA, USA) and Factera 1.4.3^37^.

### Immunohistochemistry (IHC)

The COO classification was determined by IHC using anti-CD10, MUM1, and BCL6 antibodies (Fuzhou 100 Maixin Biotech, Fuzhou, China), according to the Hans algorithm^38^. Patients were grouped into GCB or non-GCB subgroups. IHC staining was also performed for MYC, BCL2, and BCL6. Double-expressor lymphoma (DEL) was defined as MYC expression in ≥ 40% of tumor cells and BCL2 expression in ≥ 60% of tumor cells, as previously described^39^.

### Statistical analysis

Comparisons between categorical variables were performed using χ^2^-tests or Fisher’s exact tests. Refractory disease was defined as patients who achieved less than a partial response in the first-line setting or those who relapsed within the first 12 months since the initiation of front-line treatment. Progression-free survival (PFS) was calculated from the date of initial diagnosis until the first disease progression/relapse or death from any cause. Overall survival (OS) was calculated from the date of initial diagnosis until death from any cause. Survival curves were estimated using the Kaplan-Meier method and compared with the log-rank test. Multivariable Cox proportional hazard regression models were used to estimate hazard ratios for an evolving event and to identify independent prognostic factors. Comparisons between the novel prognostic model and IPI or R-IPI were performed using the C-index. The area under curve (AUC) of the time-dependent receiver operating characteristic was used to evaluate the predictive performance of models. *P* values less than 0.05 were considered as statistically significant, and all *P* values were 2-tailed. All statistical analyses were conducted using SPSS statistical software for Windows, version 26.0 (SPSS, Chicago, IL, USA) and R software, version 3.6.2 (https://www.R-project.org).

## Results

### The incidence of BCL2 genetic alterations and BCL2 protein expressions

**Figure 1:**
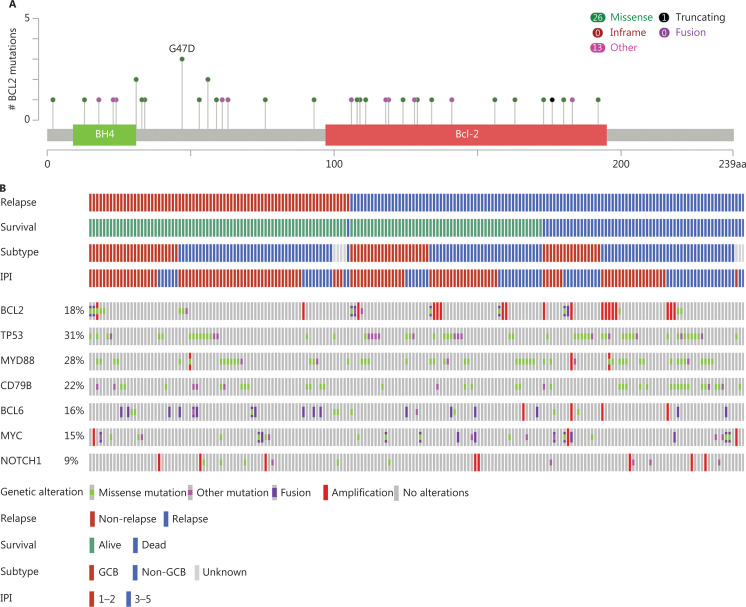
The distribution of *BCL2* and other genes in 191 DLBCL patients. DLBCL, diffuse large B-cell lymphoma; IPI, International Prognostic Index; GCB, germinal center-B cell like; non-GCB, non-germinal center-B cell like.

In total, 40 single nucleotide variants (SNVs) were identified in *BCL2* [referred to as *BCL2* mutation (*BCL2*^MUT^)] among 8.9% (17/191) of the patients, and more than half [65% (26/40)] the SNVs were missense mutations. Only 1 hotspot mutation (> 2 SNVs) with *BCL2* (G47) was identified (**Figure 1A**). *BCL2*^GA/AMP^ occurred in 9.4% (18/191) of the patients. The median threshold for *BCL2*^GA/AMP^ detection was 3.29 (range: 2.7–5.56). Additionally, only 4.2% (8/191) of the cases harbored a *BCL2* translocation (*BCL2*^TR^). As a result, *BCL2* alterations, comprised of the above 3 genetic alterations, were observed in 18.3% (35/191) of the patients. Only 1 patient (0.5%) had concurrent *BCL2*^MUT^ and *BCL2*^GA/AMP^. Out of 8 patients with *BCL2*^TR^, 7 patients had concurrent *BCL2*^MUT^, of which 3 cases had hypermutation. However, no case harbored concurrent *BCL2*^TR^ and *BCL*2^GA/AMP^. Among 171 patients with available BCL2 protein expression data, 83 (48.5%) patients were BCL2 positive, with the cutoff value of ≥ 60%. Twenty-eight out of 117 (23.9%) patients had DEL, and only 3 of 191 (1.6%) patients had DHL.

### The association between patient characteristics and BCL2 genetic alterations

Compared to those without *BCL2* alterations, patients with *BCL2* alterations were more likely to have advanced Ann Arbor stage (77.1% *vs*. 54.2%, *P* = 0.014). *BCL2*^TR^ was significantly more commonly seen in the GCB subtype (*P* = 0.026). Among those with BCL2 protein expressions, 78.1% (25/32) of the patients with *BCL2* alterations were BCL2 protein positive, whereas 41.7% (58/139) of those without *BCL2* alterations were positive (*P* < 0.001). Both the presence of *BCL2*^MUT^ and *BCL2*^GA/AMP^ were significantly associated with higher positive percentages of BCL2 protein expressions (*P* = 0.011 and *P* = 0.015, respectively). However, no significant association between *BCL2*^TR^ and BCL2 protein expression was observed (*P* = 0.267). There were significantly positive correlations of *BCL2* alterations or *BCL2*^GA/AMP^ with DEL (*P* = 0.034 and *P* = 0.039, respectively). However, no correlation was found between *BCL2*^MUT^ and DEL (*P* = 0.723). In addition, 97.1% of the patients in the *BCL2* alteration group received front-line R-CHOP or R-CHOP-like regimens, whereas 85.9% of those without an *BCL2* alteration received the abovementioned immunochemotherapy (*P* = 0.033). Detailed patient characteristics are shown in **Table 1**.

**Table 1:** Patient characteristics according to *BCL2* genetic alterations

Characteristics	Total (%)	*BCL2* alteration		*P* value	*BCL2* mutation		*P* value	*BCL2* ^GA/AMP^		*P* value
		Yes, *n* (%)	No, *n* (%)		Yes, *n* (%)	No, *n* (%)		Yes, *n* (%)	No, *n* (%)	
Age, years				0.338			0.617			0.434
Median (range)	55 (11–82)	60 (27–82)	54 (11–81)		55 (27–80)	55 (11–82)		60 (38–82)	54 (11–81)	
≥ 60	79 (41.4)	17 (48.6)	62 (39.7)		8 (47.1)	71 (40.8)		9 (50.0)	70 (40.5)	
< 60	112 (58.6)	18 (51.4)	94 (60.3)		9 (52.9)	103 (59.2)		9 (50.0)	103 (59.5)	
Gender				0.362			0.463			0.996
Male	106 (55.5)	17 (48.6)	89 (57.1)		8 (47.1)	98 (56.3)		10 (55.6)	96 (55.5)	
Female	85 (44.5)	18 (51.4)	67 (42.9)		9 (52.9)	76 (43.7)		8 (44.4)	77 (44.5)	
ECOG PS				0.535			0.377			0.999
0–1	173 (90.6)	33 (94.3)	140 (89.7)		17 (100.0)	156 (89.7)		17 (94.4)	156 (90.2)	
≥ 2	18 (9.4)	2 (5.7)	16 (10.3)		0 (0.0)	18 (10.3)		1 (5.6)	17 (9.8)
Ann Arbor stage				**0.014**			0.118			0.219
I–II	79 (41.4)	8 (22.9)	71 (45.5)		13 (76.5)	75 (43.1)		5 (27.8)	74 (42.8)	
III–IV	112 (58.6)	27 (77.1)	85 (54.2)		4 (23.5)	99 (56.9)		13 (72.2)	99 (57.2)
Primary site				0.857			0.978			0.833
Nodal	123 (64.4)	23 (65.7)	100 (64.1)		11 (64.7)	112 (64.4)		12 (66.7)	111 (64.2)	
Extranodal	68 (35.6)	12 (34.3)	56 (35.9)		6 (35.3)	62 (35.6)		6 (33.3)	62 (35.8)	
Extranodal involvement				0.856			0.250			0.734
< 2 sites	134 (70.2)	25 (71.4)	109 (69.9)		14 (82.4)	120 (69.0)		12 (66.7)	122 (70.5)	
≥ 2 sites	57 (29.8)	10 (28.6)	47 (30.1)		3 (17.6)	54 (31.0)		6 (33.3)	51 (29.5)	
COO subtype^†^				0.571			0.123			0.451
GCB	66 (34.6)	14 (40.0)	52 (33.3)		9 (52.9)	57 (32.8)		5 (27.8)	61 (35.3)	
Non-GCB	118 (61.8)	21 (60.0)	97 (62.2)		8 (47.1)	110 (63.2)		13 (72.2)	105 (60.7)	
Unknown	7 (3.7)	0 (0.0)	7 (4.5)		0 (0.0)	7 (4.0)		0 (0.0)	7 (4.0)	
IPI score				0.748			0.240			0.836
0–2	121 (63.4)	23 (65.7)	98 (62.8)		13 (76.5)	108 (62.1)		11 (61.1)	110 (63.6)	
3–5	70 (36.6)	12 (34.3)	58 (37.2)		4 (23.5)	66 (37.9)		7 (38.9)	63 (36.4)	
LDH^†^				0.837			0.695			0.695
Elevated	97 (50.8)	17 (48.6)	80 (51.3)		8 (47.1)	89 (51.1)		8 (44.4)	89 (51.4)	
Normal	91 (47.6)	17 (48.6)	74 (47.4)		9 (52.9)	82 (47.1)		9 (50.0)	82 (47.4)	
Unknown	3 (1.6)	1 (2.9)	2 (1.3)		0 (0.0)	3 (1.7)		1 (5.6)	2 (1.2)	
BCL2 IHC^†^				**< 0.001**			**0.011**			**0.015**
≥ 60%	83 (43.5)	25 (71.4)	58 (37.2)		12 (70.6)	71 (40.8)		13 (72.2)	70 (40.5)
< 60%	88 (46.1)	7 (8.0)	81 (51.9)		3 (17.6)	85 (48.9)		4 (22.2)	84 (48.6)
Unknown	20 (10.5)	3 (8.6)	17 (10.9)		2 (11.8)	18 (10.3)		1 (5.6)	19 (11.0)
MYC/BCL2 IHC^†^				**0.034**			0.723			**0.039**
DEL	28 (14.7)	10 (28.6)	18 (11.5)		3 (17.6)	25 (14.4)		7 (38.9)	21 (12.1)
Non-DEL	89 (46.6)	15 (42.9)	74 (47.4)		8 (47.1)	81 (46.6)		7 (38.9)	82 (47.4)
Unknown	74 (38.7)	10 (28.6)	64 (41.0)		6 (35.3)	68 (39.1)		4 (22.2)	70 (40.5)
First-line chemotherapy				**0.033**			0.475			0.081
R-CHOP/R-CHOP-like	164 (85.9)	34 (97.1)	130 (83.3)		16 (94.1)	148 (85.1)		18 (100.0)	146 (84.4)	
CHOP/CHOP-like	27 (14.1)	1 (2.9)	26 (16.7)		1 (5.9)	26 (14.9)		0 (0.0)	27 (15.6)	

Bold indicates significance. ECOG, Eastern Cooperative Oncology Group; PS, performance status; COO, cell of origin; GCB, germinal center-B cell like; non-GCB, non-germinal center-B cell like; IPI, International Prognostic Index; LDH, lactate dehydrogenase; IHC, immunohistochemistry; DEL, double-expressor lymphoma; GA, gain; AMP, amplification. ^†^*P* value is calculated after excluding the patients for whom the information of the corresponding variable was unknown.

### The associations between other genes and COO subtypes or IPI scores

Overall, the frequency of *TP53* mutations, *MYD88* mutations, *CD79B* mutations, *BCL6* alterations, *MYC* alterations, and *NOTCH1* alterations were 30.9% (59/191), 27.7% (53/191), 22.0% (42/191), 15.7% (30/191), 15.2% (29/191), and 9.4% (18/151), respectively (**Figure 1B**). *CD79B* mutations were seen more commonly in the non-GCB subtype (*P* = 0.039). *NOTCH1* alterations and *MYD88* mutations also tended to be more frequently found in non-GCB subtypes, but these did not reach statistical significance (*P* = 0.058 and *P* = 0.057, respectively). However, *MYC* alterations showed a trend, occurring more frequently in the GCB subtype (*P* = 0.082). Additionally, there was no significant difference between the IPI risk groups and these genes. The correlations of these genes with COO subtypes or IPI are shown in **Figure 1B** and detailed in **Supplementary Table S1**.

### The associations between BCL2 genetic alterations and other genes

The associations of *BCL2* alterations, *BCL2*^MUT^, and *BCL2*^GA/AMP^ with other genes were analyzed. The presence of *BCL2* alterations and *BCL2*^MUT^ tended to be positively associated with *MYD88* mutations, but the difference did not reach statistical significance (*P* = 0.072 and *P* = 0.089, respectively). No significant correlation was found between *BCL2* genetic alterations and all other genes (**Supplementary Table S2**).

### Survival analysis of DLBCL patients treated with R-CHOP

#### Impact of BCL2 genetic variations on survival outcomes

**Figure 2:**
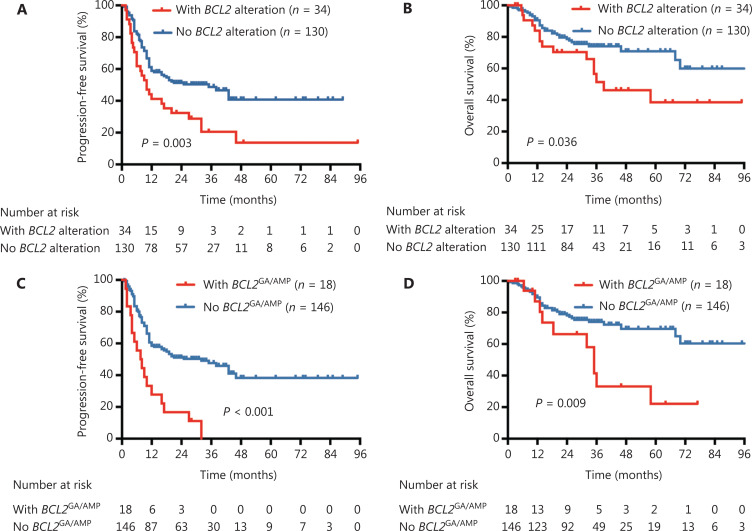
Survival stratified by *BCL2* genetic alterations in 164 DLBCL patients receiving R-CHOP/R-CHOP-like regimens. (A) PFS stratified by *BCL2* alteration; (B) OS stratified by *BCL2* alteration; (C) PFS stratified by *BCL2*^GA/AMP^; (D) OS stratified by *BCL2*^GA/AMP^. DLBCL, diffuse large B-cell lymphoma; PFS, progression-free survival; OS, overall survival; GA, gain; AMP, amplification.

Overall, 164 patients treated with R-CHOP or R-CHOP-like regimens were included for survival and prognostic analyses. With a median follow-up of 35 months (range: 1–118 months), 48 deaths occurred. For all 164 patients, the 5-year PFS and 5-year OS were 34.0% and 62.0%, respectively. Compared with patients with the absence of *BCL2* alterations, the 5-year PFS (13.7% *vs.* 40.8%, *P* = 0.003) and OS (34.0% *vs.* 70.9%, *P* = 0.036) were significantly decreased in cases that harbored *BCL2* alterations (**Figure 2A–B**). Importantly, patients who harbored *BCL2*^GA/AMP^ also had a remarkably poorer PFS (5-year PFS, 11.1% *vs.* 38.3%, *P* < 0.001) and OS (5-year OS, 22.1% *vs.* 69.6%, *P* = 0.009) compared with those without *BCL2*^GA/AMP^ (**Figure 2C–D**). In contrast, neither *BCL2*^MUT^ nor *BCL2*^TR^ were significantly prognostic for the PFS and OS.

#### Univariable analysis for PFS and OS

In addition to *BCL2* alterations and *BCL2*^GA/AMP^, univariate analyses also showed that age > 60 years (PFS, *P* = 0.033; OS, *P* = 0.009), ECOG PS of ≥ 2 (PFS, *P* = 0.009; OS, *P* < 0.001), advanced stage (PFS, *P* < 0.001; OS, *P* < 0.001), number of extranodal involvement sites ≥ 2 (PFS, *P* = 0.013; OS, *P* < 0.001), elevated LDH (PFS, *P* = 0.001; OS, *P* < 0.001), IPI score of 3–5 (PFS, *P* < 0.001; OS, *P* < 0.001) (**Supplementary Figure S2**), and *TP53* mutation (PFS, *P* = 0.014; OS, *P* = 0.047) (**Supplementary Figure S2**) were all significantly associated with poor survival. The presence of *MYC* translocations (*P* = 0.062) and *CD79B* mutations (*P* = 0.066) tended to have negative prognostic effects on the OS, but without statistical significance. The results of univariate analysis for the PFS and OS are summarized in **Table 2**.

**Table 2:** Univariable analysis for PFS and OS in patients receiving R-CHOP/R-CHOP-like regimens

Risk factors	PFS		OS	
	5-year PFS rate (%)	*P* value	5-year OS rate (%)	*P* value
Age, years (≥ 60 *vs.* < 60)	17.1 *vs.* 46.8	**0.033**	48.2 *vs.* 76.9	**0.009**
Gender (male *vs.* female)	35.4 *vs.* 32.7	0.687	63.9 *vs.* 60.0	0.450
ECOG PS (0–1 *vs.* ≥ 2)	35.2 *vs.* 29.4	**0.009**	65.5 *vs.* 31.9	**< 0.001**
Ann Arbor stage (I–II *vs.* III–IV)	56.2 *vs.* 20.6	**< 0.001**	82.5 *vs.* 49.2	**< 0.001**
COO subtype (GCB *vs.* non-GCB)	30.2 *vs.* 34.9	0.738	65.4 *vs.* 61.2	0.533
Primary site (nodal *vs.* extranodal)	31.7 *vs.* 45.0	0.771	63.0 *vs.* 62.2	0.539
Extranodal involvement (< 2 *vs.* ≥ 2 sites)	38.6 *vs.* 22.8	**0.013**	72.7 *vs.* 38.7	**< 0.001**
LDH (elevated *vs.* normal)	25.6 *vs.* 46.0	**0.001**	47.6 *vs.* 80.8	**< 0.001**
IPI score (0–2 *vs.* 3–5)	45.3 *vs.* 18.6	**< 0.001**	76.6 *vs.* 39.5	**< 0.001**
BCL2 IHC (≥ 60% *vs.* < 60%)	28.0 *vs.* 31.1	0.143	64.5 *vs.* 60.0	0.856
DEL (yes *vs.* no)	38.6 *vs.* 39.5	0.361	52.1 *vs.* 66.6	0.595
*TP53* mutation (yes *vs.* no)	29.8 *vs.* 35.5	**0.014**	53.4 *vs.* 66.3	**0.047**
*BCL2* alteration (yes *vs.* no)	13.7 *vs.* 40.8	**0.003**	38.5 *vs.* 70.9	**0.036**
*BCL2* mutation (yes *vs.* no)	35.1 *vs.* 31.3	0.67	67.0 *vs.* 61.4	0.671
*BCL2*^GA/AMP^ (yes *vs.* no)	0 *vs.* 38.3	**< 0.001**	22.1 *vs.* 69.6	**0.009**
*BCL2* translocation (yes *vs.* no)	0 *vs.* 36.7	0.116	68.6 *vs.* 61.8	0.778
*MYC* alteration (yes *vs.* no)	33.5 *vs.* 34.3	0.501	42.8 *vs.* 66.6	0.137
*MYC* mutation (yes *vs.* no)	39.7 *vs.* 33.1	0.845	65.2 *vs.* 61.4	0.804
*MYC* translocation (yes *vs.* no)	18.5 *vs.* 35.1	0.097	21.0 *vs.* 66.6	0.062
*NOTCH1* alteration (yes *vs.* no)	47.1 *vs.* 32.4	0.813	67.2 *vs.* 60.9	0.398
*MYD88* mutation (yes *vs.* no)	13.7 *vs.* 39.9	0.094	53.3 *vs.* 66.7	0.271
*MYD88* L265P mutation (yes *vs.* no)	34.4 *vs.* 34.8	0.812	73.7 *vs.* 61.2	0.807
*MYD88* other mutation (yes *vs.* no)	13.9 *vs.* 38.3	0.126	48.6 *vs.* 67.0	0.339
*CD79B* mutation (yes *vs.* no)	39.2 *vs.* 32.0	0.978	53.4 *vs.* 64.6	0.066
*BCL6* alteration (yes *vs.* no)	41.6 *vs.* 34.4	0.195	67.9 *vs.* 61.6	0.397
*BCL6* translocation (yes *vs.* no)	66.7 *vs.* 30.6	0.094	85.6 *vs.* 60.0	0.219
*BCL6* mutation (yes *vs.* no)	34.2 *vs.* 52.5	0.116	87.5 *vs.* 59.7	0.178

Bold indicates significance. PFS, progression-free survival; OS, overall survival; ECOG, Eastern Cooperative Oncology Group; PS, performance status; COO, cell of origin; IPI, International Prognostic Index; LDH, lactate dehydrogenase; IHC, immunohistochemistry; DEL, double-expressor lymphoma; GA, gain; AMP, amplification.

#### The prognostic effects of BCL2 alterations and BCL2^GA/AMP^ within IPI and TP53 mutations

**Figure 3:**
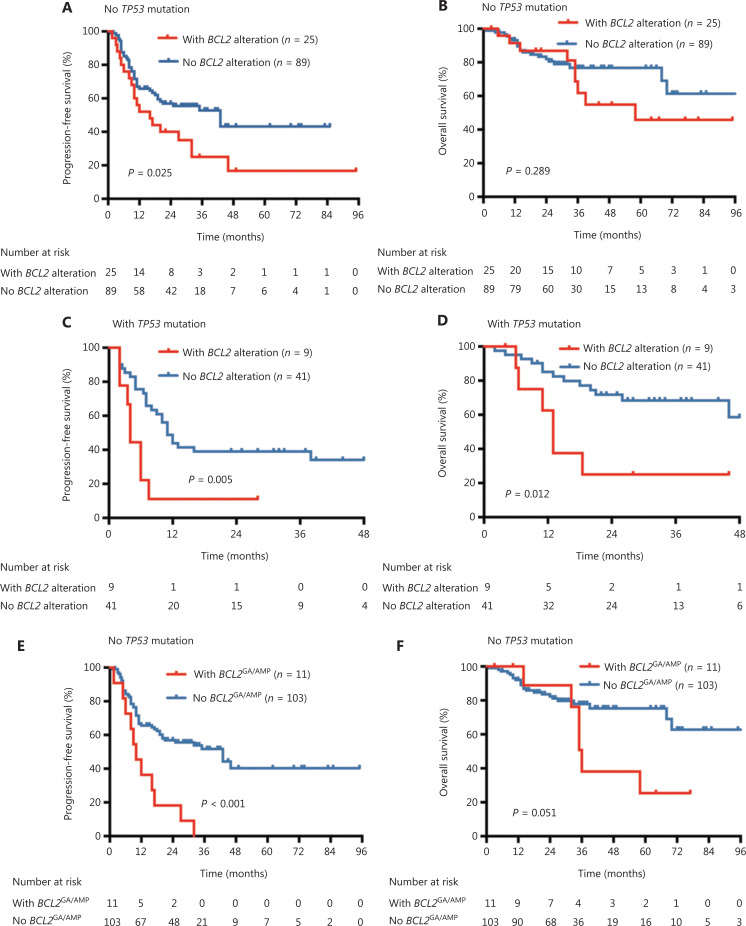

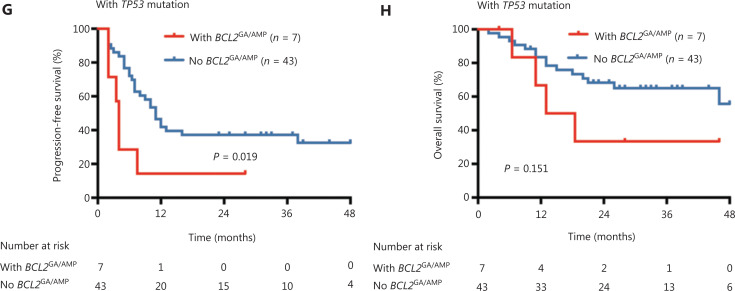
Survival stratified by *BCL2* genetic alterations in patients with or without *TP53* mutations. (A) PFS stratified by *BCL2* alteration in patients without *TP53* mutations. (B) OS stratified by *BCL2* alterations in patients without *TP53* mutations. (C) PFS stratified by *BCL2* alterations in patients with *TP53* mutations. (D) OS stratified by *BCL2* alterations in patients with *TP53* mutations. (E) PFS stratified by *BCL2*^GA/AMP^ in patients without *TP53* mutations. (F) OS stratified by *BCL2*^GA/AMP^ in patients without *TP53* mutations. (G) PFS stratified by *BCL2*^GA/AMP^ in patients with *TP53* mutations. (H) OS stratified by *BCL2*^GA/AMP^ in patients with *TP53* mutations. PFS, progression-free survival; OS, overall survival; GA, gain; AMP, amplification.

The prognostic power of *BCL2* alterations seemed to be similar between 2 IPI risk subgroups, whereas a significant association of *BCL2*^GA/AMP^ with survival outcomes was found in patients with an IPI score of 1–2 (**Supplementary Figure S3**), probably due to the small number of patients with *BCL2*^GA/AMP^ in the IPI score 3–5 subgroup. The impact of *BCL2* alterations and *BCL2*^GA/AMP^ on PFS and OS in patients either with or without *TP53* mutations were similar to that in the entire patient cohort (**Figure 3**).

Notably, 9 patients who harbored concurrent *BCL2* alterations and *TP53* mutations had a very poor prognosis, with the median PFS of only 4 months and OS of 13 months. Eight out of these 9 patients were primary refractory to first-line R-CHOP regimens, whereas only 1 case (Case 47) remained disease progression free at the last follow-up (**Supplementary Table S3**). Case 47 was a 38-year-old male, diagnosed with stage IE primary testicular DLBCL. He underwent radical orchiectomy, and then received R-CHOP chemotherapy, followed by prophylactic irradiation to the contralateral testis. This patient had a PFS of 28 months as of August 2020.

### Independent prognostic factors for DLBCL patients treated with R-CHOP

Considering that the IPI involved age, ECOG PS, number of extranodal involvement site, LDH and Ann Arbor stage, these 5 prognostic indicators, though statistically significant in univariate analyses, were not incorporated into further multivariable analyses. In a multivariable analysis for PFS that incorporated *BCL2* alterations, *TP53* mutations, and the IPI, *BCL2* alterations [hazard ratio (HR): 2.519; 95% confidence interval (CI): 1.586–4.001; *P* < 0.001], *TP53* mutation (HR: 2.055; 95% CI: 1.334–3.167; *P* = 0.001), and IPI (HR: 2.479; 95% CI: 1.641–3.746; *P* < 0.001) were independent factors predicting PFS (**Table 3**). Similarly, in a multivariable analysis incorporating *BCL2*^GA/AMP^, *TP53* mutations, and IPI, *BCL2*^GA/AMP^ (HR: 3.074; 95% CI: 1.801–5.246; *P* < 0.001) remained an independent prognostic factor for PFS, in addition to *TP53* mutations and the IPI. Regarding OS, multivariable analyses, including *BCL2* alterations, *TP53* mutations, and IPI, showed that *BCL2* alterations (HR: 2.610; 95% CI: 1.391–4.896; *P* = 0.003), *TP53* mutations (HR: 2.295; 95% CI: 1.263–4.170; *P* = 0.006) and IPI (HR: 4.068; 95% CI: 2.236–7.401; *P* < 0.001) were significant predictors of OS. The presence of *BCL2*^GA/AMP^ also showed a high degree of correlation with OS (HR: 2.586; 95% CI: 1.279–5.232; *P* = 0.008), independent of *TP53* mutations and the IPI.

**Table 3:** Multivariable analysis for PFS and OS in patients receiving R-CHOP/R-CHOP-like regimens

Model and variables	PFS		OS		
	HR (95% CI)	*P* value	HR (95% CI)	*P* value	Score
Model 1					
*BCL2* alteration (yes *vs.* no)	2.519 (1.586–4.001)	**< 0.001**	2.610 (1.391–4.896)	**0.003**	1 *vs.* 0
*TP53* mutation (yes *vs.* no)	2.055 (1.334–3.167)	**0.001**	2.295 (1.263–4.170)	**0.006**	1 *vs.* 0
IPI score (3–5 *vs.* 1–2)	2.479 (1.641–3.746)	**< 0.001**	4.068 (2.236–7.401)	**< 0.001**	2 *vs.* 0
Model 2					
*BCL2*^GA/AMP^ (yes *vs.* no)	3.074 (1.801–5.246)	**< 0.001**	2.586 (1.279–5.232)	**0.008**	1 *vs.* 0
*TP53* mutation (yes *vs.* no)	1.901 (1.239–2.918)	**0.003**	2.138 (1.182–3.870)	**0.012**	1 *vs.* 0
IPI score (3–5 *vs.* 1–2)	2.266 (1.503–3.415)	**< 0.001**	3.750 (2.067–6.800)	**< 0.001**	2 *vs.* 0

Bold indicates significance. PFS, progression-free survival; OS, overall survival; IPI, International Prognostic Index; HR, hazard ratio; CI, confidence interval; GA, gain; AMP, amplification.

### A novel prognostic model for OS in DLBCL patients treated with R-CHOP

**Figure 4:**
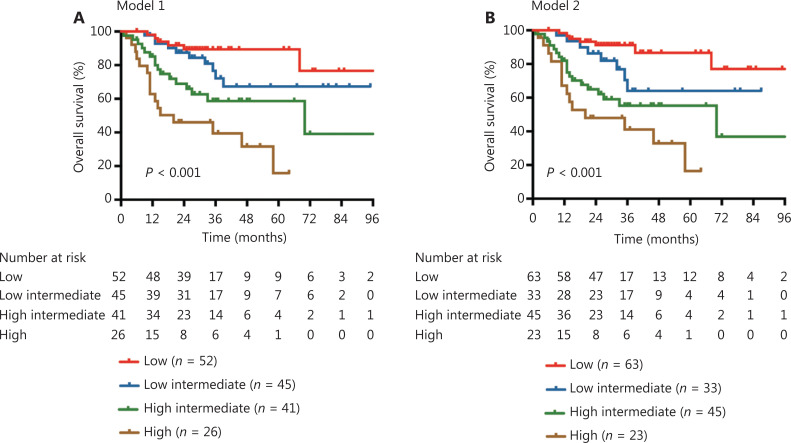
Survival outcomes stratified by prognostic models. (A) Overall survival (OS) stratified by Model 1. (B) OS stratified by Model 2.

Based on the prognostic factors derived from the multivariable analyses and corresponding HRs, novel prognostic risk models for OS were proposed. Accordingly, the adopted weights of each adverse prognostic factor were as follows: 1 point for each risk factor *BCL2* alteration (Model 1) or *BCL2*^GA/AMP^ (Model 2), or TP53 mutation; and 2 points for the IPI score ≥ 3 (**Table 3**). Patients were further stratified into 4 risk groups based on their scores (low risk, 0 point; low-intermediate risk, 1 point; high-intermediate risk, 2 points; high risk, 3–4 points). In Model 1, 52 (31.7%), 45 (27.4%), 41 (25.0%), and 26 (15.9%) patients were classified into the low risk, low-intermediate risk, high-intermediate risk, and high risk groups, respectively. There were significant differences in survival outcomes among these 4 risk groups, with a 5-year OS of 89.4%, 67.3%, 58.7%, and 15.8% (*P* < 0.001), respectively, for the 4 risk groups (**Figure 4A**). According to Model 2, the distribution of 164 patients were as follows: the low risk, 63 (38.4%) patients; the low-intermediate risk, 33 (20.1%) patients; the high-intermediate risk, 45 (27.4%), and the high risk group, 23 (14.0%) patients. The 5-year OS rates were 86.6%, 64.1%, 55.3%, and 16.4% for the 4 risk groups (*P* < 0.001), respectively (**Figure 4B**).

The new prognostic models also showed better levels of accuracy for predicting OS than both the IPI and R-IPI, with a C-index of 0.715 for Model 1 and 0.722 for Model 2, when compared with that of 0.664 for the IPI and 0.693 for the R-IPI. Additionally, the AUC for predicting the 5-year OS of Model 1 (0.781) and Model 2 (0.790) were higher than that of IPI (0.697) and R-IPI (0.725) (**Supplementary Figure S4**).

## Discussion

In this study involving patients with *de novo* DLBCL uniformly treated with R-CHOP/R-CHOP-like regimens, the methodology of probe capture-based high-resolution sequencing was used to analyze the relationships of genetic alterations with clinical outcomes. Our findings showed that the presence of *BCL2* alterations, especially *BCL2*^GA/AMP^ and *TP53* mutations were significantly associated with inferior outcomes, and were independent of the IPI. Therefore, we proposed novel prognostic models that incorporated both clinical and genetic characteristics. The models were based on 3 risk factors, including *BCL2* alterations (Model 1) or *BCL2*^GA/AMP^ (Model 2), combined with *TP53* mutations and IPI, identifying 4 risk groups with different survival outcomes. With the emergence of targeted therapy, such as BCL2 inhibitors or immune checkpoint inhibitors, such prognostic models with biomarkers could aid in further defining the patients suitable for alternative treatment approaches and/or enrollment in clinical trials.

The prognostic significance of BCL2 protein expression and gene aberrations has been reported in a great number of studies, but with conflicting findings^22–25,40–44^. The percentage of high BCL2 expression in DLBCL, with the cutoff value of 70%, ranged between 40%–50%^22,23,40,41,45^. BCL2 high expression has been found to be a strong predictor of poor prognosis in some studies^23,40^, but not all studies^41,45^. Thereafter, it is believed that double expression of MYC and BCL2 protein contributes to inferior outcomes^22,46^, nevertheless, there is still controversy^40^. Interestingly, a study showed that DEL was significantly associated with inferior survival within the GCB subgroup, but not within the ABC subgroup^46^. In this study, neither BCL2 high expression alone, nor co-expression with MYC predicted poor survival, consistent with previous reports^41,45^. The finding that BCL2 protein expression may be less relevant to outcome should be further confirmed.

Also, the prognostic significance of the *BCL2* translocation t (14;18) in DLBCL has not yet been fully investigated. Some studies showed no prognostic implication of *BCL2*^TR23,24,47^, whereas others demonstrated adverse prognostic significance in GCB subtype independent of the IPI^26,43^. The worse outcome associated with *BCL2*^TR^ might be related to the second hit of *MYC* translocation^27^. In this study, the incidence of *BCL2*^TR^ was only 4.2% (7/191), which was lower than that reported in previous studies^23,26^, probably owing to different methodologies used across studies. Our results also showed that *BCL2*^TR^ was not associated with survival, and the analysis for DHL was not conducted due to the relative rarity of cases. Further studies with larger numbers of patients are needed to confirm these observations.

Unlike extensive studies on BCL2 expression and *BCL2*^TR^, there are few reports on *BCL2*^GA/AMP^ and *BCL2*^MUT^. Recently, 2 studies with large cohorts reported that *BCL2*^GA/AMP^ was independently associated with poor outcomes in DLBCL patients^26,48^, and the prognostic power was particularly observed in ABC subtype^26^. Schuetz et al.^28^ reported that the *BCL2*^MUT^ was not independently associated with survival. In the present study, we focused on the prognostic effects of *BCL2*^GA/AMP^ and *BCL2*^MUT^, which were not concurrently examined in the overwhelming majority of previous studies. Our analyses showed that *BCL2* alterations significantly correlated with inferior survival, but it was noteworthy that only *BCL2*^GA/AMP^ predicted a poor prognosis, rather than *BCL2*^MUT^ or *BCL2*^TR^, which was in accordance with previous findings^26,28,48^. In addition, consistent with recent studies^49,50^, we also found that positive BCL2 expression was significantly associated with *BCL2*^GA/AMP^, indicating the BCL2 expression was partly driven by CNV.

We speculate that the inconsistent results obtained by different studies regarding the effect of *BCL2* on prognoses for DLBCL, may be partly attributable to the complexity of *BCL2* genetic alterations. Another reason may be that the testing methods most studies used were not able to include all genetic alterations. By using probe capture-based high resolution sequencing, we simultaneously obtained comprehensive information about *BCL2* genetic alterations, including mutations, gain/amplifications, and translocations. In this study, *BCL2*^TR^ was frequently accompanied by *BCL2*^MUT^ (7/8 cases), sometimes hypermutation (3/7 cases), whereas *BCL2*^GA/AMP^ cases rarely were characterized with concurrent *BCL2*^MUT^ (1/18 cases). This phenomenon confirmed the previous finding that *BCL2*^TR^ played a pivotal role in the acquisition of *BCL2*^MUT^, and *BCL2*^MUT^ likely occurred as a result of aberrant somatic hypermutations^28^. Some *BCL2* mutations, especially hypermutations, may partly influence BCL2 protein functions^51^. Because *BCL2*^TR^ is frequently accompanied by *BCL2*^MUT^, and the effect of *BCL2*^TR^ on the function of BCL2 protein is unpredictable, these characteristics could partly explain the inconsistent prognostic results of *BCL2*^TR^. In contrast, *BCL2*^GA/AMP^ is rarely accompanied by *BCL2*^MUT^, resulting in high expression of BCL2 protein whose function has not been affected, thus enhancing the anti-apoptotic ability of tumor cells. Taken together, the biological basis of the association between *BCL2* genetic alterations and prognoses remains unclear, so further investigations are needed to elucidate the responsible mechanisms.

The mutation percentage of *TP53* in DLBCL is approximately 20%–25%^13,14,52^. In this study, the incidence was as high as 31%, possibly due to the preference for relapsed and refractory subsets in patient selection. Cumulative studies have shown that *TP53* mutations were significantly associated with a lower rate of complete remission and shorter PFS and OS in patients with DLBCL treated with either CHOP^52^ or R-CHOP regimens^13,14^. In the present study, *TP53* mutations were also identified as an independent factor predicting poor outcomes, in agreement with previous reports. *TP53* mutations could potentially provide predictive information to guide precise treatment for patients with DLBCL.

Both *BCL2* and *TP53* have been shown to play a central role in the inhibition of apoptosis and tumor suppression. In this study, among 9 patients with concurrent *BCL2* alterations and *TP53* mutations, 8 were primary refractory to first-line R-CHOP regimens. Despite the small number of patients, the incorporation of *BCL2* alterations and *TP53* mutations could define a subset of cases with an extremely poor prognosis. This phenomenon might further reflect the crucial role of the inactivated anti-apoptotic pathway in rendering B-cells resistant to standard immunochemotherapy. From this perspective, therapeutic approaches making the anti-apoptotic pathway activated, such as inhibition of *BCL2*, might confer reversal of drug-resistance and further improve survival outcomes of patients with DLBCL treated with R-CHOP. Venetoclax, a highly selective BCL2 inhibitor, plus R-CHOP in a first-line setting, have demonstrated promising antitumor activities in DLBCL patients^53,54^. However, more studies are required to provide definitive evidence.

The novel prognostic models were constructed using clinical and genetic characteristics involving *BCL2* alterations (Model 1), *BCL2*^GA/AMP^ (Model 2), *TP53* mutations, and the IPI. To the best of our knowledge, no study using these 3 combined indicators for individual risk prediction has been reported. The prognostic models incorporating both genetic and clinical information are important for risk stratification, and also have significant therapeutic implications, which may aid physicians in making clinical decisions. For those high risk patients defined by the novel models, the 5-year OS was only approximately 16%, thus novel therapeutic strategies, including new targeted therapy, immune checkpoint inhibitors, or additional therapies are needed for better efficacy. Despite these challenges, further external validation of the novel prognostic models is still warranted.

Several limitations to this study need to be acknowledged. This study was limited by its retrospective nature, which could inevitably have caused bias during patient selection and subsequent study processes. Another limitation was the lack of validation of the novel prognostic models in an independent cohort. In addition, the sample size was relatively small in several patient subgroups, which limited the power of our analysis. For instance, with only 8 cases harboring *BCL2*^TR^, the finding that *BCL2*^TR^ was not significantly correlated with BCL2 expression and survival should be further confirmed. In spite of these limitations, this study provided important insight into individual risk assessments, and provided the basis for future investigations.

In conclusion, this study comprehensively described genetic alterations of *BCL2*. In patients treated with R-CHOP or R-CHOP-like regimens, the presence of *BCL2* alterations, especially *BCL2*^GA/AMP^, and *TP53* mutations were significantly associated with poor outcomes, independent of the IPI. We proposed a novel prognostic model based on 3 risk factors, including *BCL2* alterations (Model 1), *BCL2*^GA/AMP^ (Model 2), *TP53* mutations, and the IPI, which identified 4 risk groups with different survival outcomes. Once the new prognostic models have been validated in an independent cohort, the models will help to further define DLBCL patients with poor prognoses, who were treated with R-CHOP, and will identify patients suitable for alternative treatment approaches and/or enrollment in clinical trials.
