# Supplementary material for: Prognostic value of BCL2 and TP53 genetic alterations for diffuse large B-cell lymphoma patients treated with R-CHOP
Source: Cancer Biol Med. 2021 Nov 23;19(6):893–909. doi: 10.20892/j.issn.2095-3941.2021.0193 (PMC9257313; doi:10.20892/j.issn.2095-3941.2021.0193)
Supplement: Supplementary file 1 [file cbm-19-893-s001.pdf]

Supplementary materials

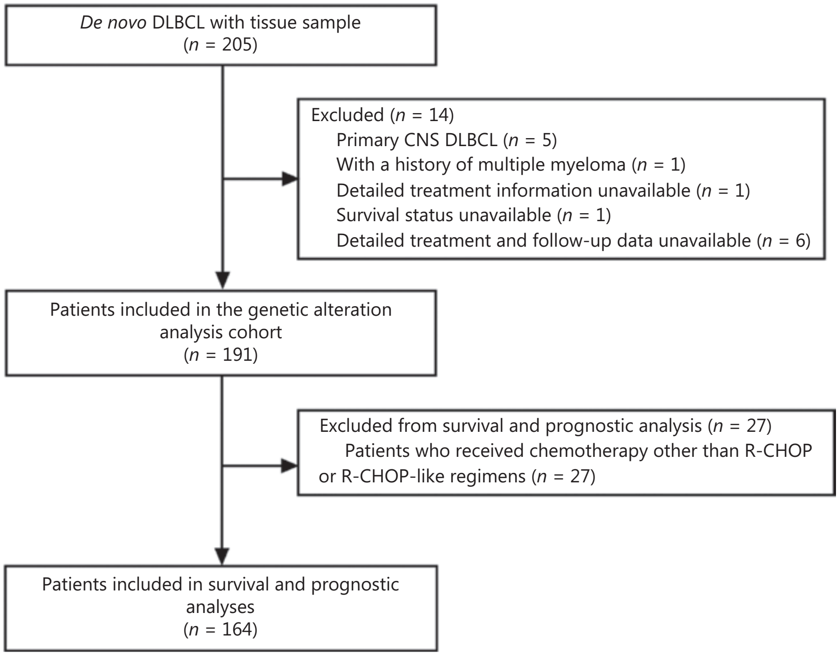

Figure S1 The patient selection process. DLBCL, diffuse large B-cell lymphoma; CNS, central nervous system.

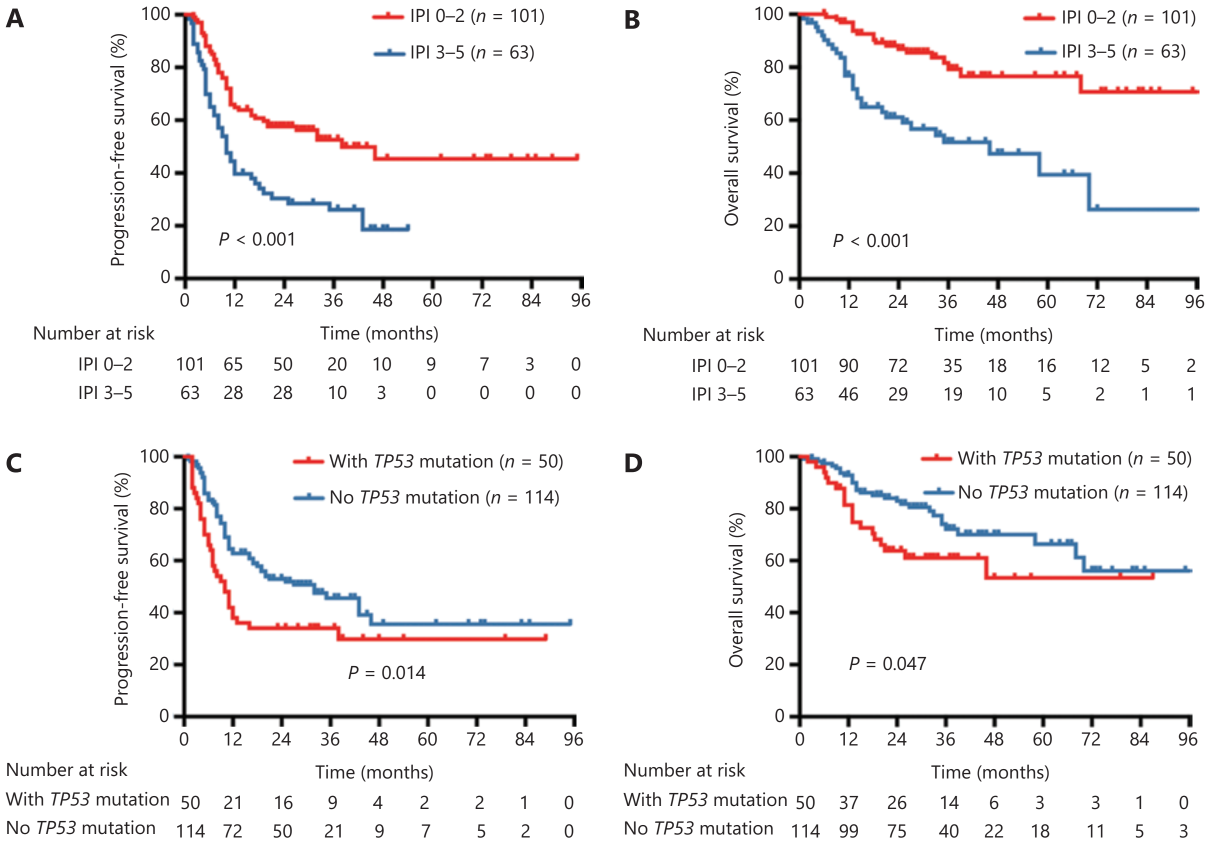

Figure S2 Survival stratified by the IPI and *TP53* mutations in 164 DLBCL patients receiving R-CHOP/R-CHOP-like regimens. (A) PFS stratified by the IPI. (B) OS stratified by the IPI. (C) PFS stratified by *TP53* mutations. (D) OS stratified by *TP53* mutations. DLBCL, diffuse large B-cell lymphoma; PFS, progression-free survival; OS, overall survival; IPI, International Prognostic Index.

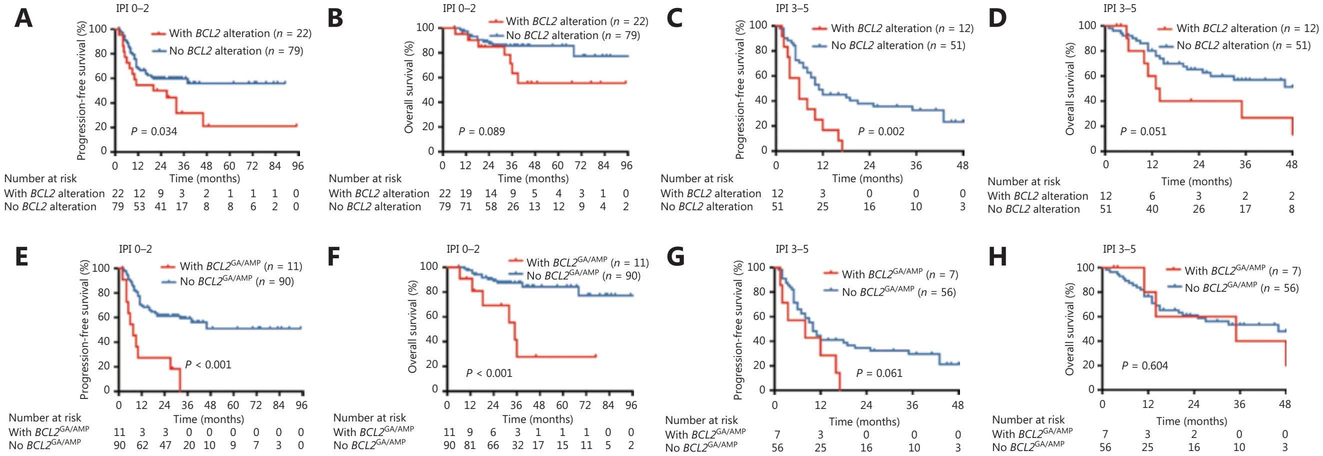

Figure S3 Survival stratified by *BCL2* genetic alterations in each IPI risk group. (A) PFS stratified by *BCL2* alterations in patients with IPI scores of 0–2. (B) OS stratified by *BCL2* alterations in patients with IPI scores of 0–2. (C) PFS stratified by *BCL2* alterations in patients with IPI scores of 3–5. (D) OS stratified by *BCL2* alterations in patients with IPI scores of 3–5. (E) PFS stratified by *BCL2*$^{GA/AMP}$ in patients with IPI scores of 0–2. (F) OS stratified by *BCL2*$^{GA/AMP}$ in patients with IPI scores of 0–2. (G) PFS stratified by *BCL2*$^{GA/AMP}$ in patients with IPI scores of 3–5. (H) OS stratified by *BCL2*$^{GA/AMP}$ in patients with IPI scores of 3–5. IPI, International Prognostic Index; PFS, progression-free survival; OS, overall survival; GA, gain; AMP, amplification.

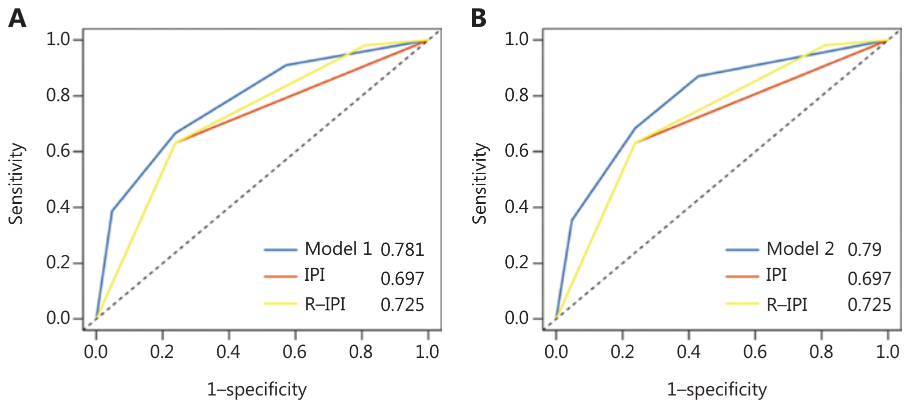

Figure S4 Comparison of the new prognostic models with IPI and R-IPI. (A) The AUC for predicting the 5-year OS of Model 1, IPI, and R-IPI. (B) The AUC for predicting the 5-year OS of Model 2, IPI, and R-IPI. IPI, International Prognostic Index; R-IPI, revised International Prognostic Index; AUC, the area under curve; OS, overall survival.

Table S1 The correlations of several genes with COO subtype or IPI score

Genetic alterations	COO subtype		*P* value	IPI score		*P* value
	GCB, *n* (%)	Non-GCB, *n* (%)		0–2, *n* (%)	3–5, *n* (%)	
TP53 mutation			0.94			0.674
Yes	16 (30.2)	32 (30.8)		32 (31.7)	18 (28.6)	
No	37 (69.8)	72 (69.2)		69 (68.3)	45 (71.4)	
NOTCH1 alteration			0.058			0.183
Yes	2 (3.8)	14 (13.5)		13 (12.9)	4 (6.3)	
No	51 (96.2)	90 (86.5)		88 (87.1)	59 (93.7)	
MYD88 mutation			0.057			0.366
Yes	11 (20.8)	37 (35.6)		27 (26.7)	21 (33.3)	
No	42 (79.2)	67 (64.4)		74 (73.3)	42 (66.7)	
CD79B mutation						0.066
Yes	7 (13.2)	29 (27.9)	**0.039**	18 (17.8)	19 (30.2)	
No	46 (86.8)	75 (72.1)		83 (82.2)	44 (69.8)	
MYC alteration			0.082			0.596
Yes	13 (24.5)	14 (13.5)		16 (15.8)	12 (19.0)	
No	40 (75.5)	90 (86.5)		85 (84.2)	51 (81.0)	
BCL6 alteration			0.910			0.533
Yes	8 (15.1)	15 (14.4)		14 (13.9)	11 (17.5)	
No	45 (84.9)	89 (85.6)		87 (86.1)	52 (82.5)	

Bold indicates significance. COO, cell of origin; IPI, International Prognostic Index.

Table S2 Correlation between *BCL2* and other genes

Characteristic	Total (%)	*BCL2* alteration		*P* value	*BCL2* mutation		*P* value	$BCL2^{GA/AMP}$		*P* value
		Yes, *n* (%)	No, *n* (%)		Yes, *n* (%)	No, *n* (%)		Yes, *n* (%)	No, *n* (%)	
TP53 mutation				0.743			0.491			0.440
Yes	59 (30.9)	10 (28.6)	49 (31.4)		4 (23.5)	55 (31.6)		7 (38.9)	52 (30.1)	
No	132 (69.1)	25 (71.4)	107 (68.6)		13 (76.5)	119 (68.4)		11 (61.1)	121 (69.9)	
NOTCH1 alteration				0.204			0.377			1
Yes	18 (9.4)	1 (2.9)	17 (10.9)		0 (0.0)	18 (10.3)		1 (5.6)	17 (9.8)	
No	173 (90.6)	34 (97.1)	139 (89.1)		17 (100.0)	156 (89.7)		17 (94.4)	156 (90.2)	
MYD88 mutation				0.073			0.086			0.277
Yes	53 (27.7)	14 (40.0)	39 (25.0)		8 (47.1)	45 (25.9)		7 (38.9)	46 (26.6)	
No	138 (72.3)	21 (60.0)	117 (75.0)		9 (52.9)	129 (74.1)		11 (61.1)	127 (73.4)	
MYD88 L265P mutation				0.767			0.395			0.999
Yes	20 (10.5)	4 (11.4)	16 (10.3)		3 (17.6)	17 (9.8)		2 (11.1)	18 (10.4)	
No	171 (89.5)	31 (88.6)	140 (89.7)		14 (82.4)	157 (90.2)		16 (88.9)	155 (89.6)	
MYD88 other mutation				0.065			0.192			0.326
Yes	34 (17.8)	10 (28.6)	24 (15.4)		5 (29.4)	29 (16.7)		5 (27.8)	29 (16.8)	
No	157 (82.2)	25 (71.4)	132 (84.6)		12 (70.6)	145 (83.3)		13 (72.2)	144 (83.2)	
CD79B mutation				0.891			0.999			0.553
Yes	42 (22.0)	8 (22.9)	34 (21.8)		4 (23.5)	38 (21.8)		5 (27.8)	37 (21.4)	
No	149 (78.0)	27 (77.1)	122 (78.2)		13 (76.5)	136 (78.2)		13 (72.2)	136 (78.6)	
MYC alteration				0.380			0.727			0.740
Yes	29 (15.2)	7 (20.0)	22 (14.1)		3 (17.6)	26 (14.9)		3 (16.7)	26 (15.0)	
No	162 (84.8)	28 (80.0)	134 (85.9)		14 (82.4)	148 (85.1)		15 (83.3)	147 (85.0)	
MYC mutation				1			0.694			0.699
Yes	20 (10.5)	3 (8.6)	17 (10.9)		2 (11.8)	18 (10.3)		1 (5.6)	19 (11.0)	
No	171 (89.5)	32 (91.4)	139 (89.1)		15 (88.2)	156 (89.7)		17 (94.4)	154 (89.0)	
MYC translocation				0.262			0.325			0.352
Yes	13 (6.8)	4 (11.4)	9 (5.8)		2 (11.8)	11 (6.3)		2 (11.1)	11 (6.4)	
No	178 (93.2)	31 (88.6)	147 (94.2)		15 (88.2)	163 (93.7)		16 (88.9)	162 (93.6)	
BCL6 alteration				0.796			0.734			1
Yes	30 (15.7)	6 (17.1)	24 (15.4)		3 (17.6)	27 (15.5)		3 (16.7)	27 (15.6)	
No	161 (84.3)	29 (82.9)	132 (84.6)		14 (82.4)	147 (84.5)		15 (83.3)	146 (84.4)	
BCL6 translocation				0.743			0.652			0.377
Yes	17 (8.9)	2 (5.7)	15 (9.6)		2 (11.8)	15 (8.6)		0 (0.0)	17 (9.8)	
No	174 (91.1)	33 (94.3)	141 (90.4)		15 (88.2)	159 (91.4)		18 (100.0)	156 (90.2)	
BCL6 mutation				0.692			1			0.604
Yes	11 (5.8)	1 (2.9)	10 (6.4)		1 (5.9)	10 (5.7)		0 (0.0)	11 (6.4)	
No	180 (94.2)	34 (97.1)	146 (93.6)		16 (94.1)	164 (94.3)		18 (100.0)	162 (93.6)	

GA, gain; AMP, amplification.

Table S3 Characteristics of patients with concurrent *BCL2* alterations and *TP53* mutations

ID	Age	Gender	Primary site	COO subtype	IPI	*BCL2* alteration	*TP53* mutation	First-line treatment	Response	PFS (mon)
Case 16	44	Male	Intranodal	non-GCB	3	*BCL2*^{MUT}	p.V216	R-CHOP-like	PR	6
Case 47	38	Male	Testis	GCB	0	*BCL2*^{MUT}	p.H179L	Surgery+R-CHOP-like+Radiotherapy	CR	28
Case 56	73	Female	Intranodal	non-GCB	4	*BCL2*^{MUT}	p.F134L	R-CHOP	PD	6
Case 64	63	Female	Intranodal	non-GCB	3	*BCL2*$^{GA/AMP}$	p.R248Q	R-CHOP	PD	2
Case 70	61	Female	Intranodal	non-GCB	1	*BCL2*$^{GA/AMP}$	Missing	R-CHOP	PD	2
Case 78	39	Male	Intranodal	non-GCB	2	*BCL2*$^{GA/AMP}$	p.C135fs	R-CHOP	PD	4
Case 107	44	Female	Intranodal	non-GCB	0	*BCL2*$^{GA/AMP}$	p.R282W	R-CHOP-like	PD	4
Case 135	62	Male	Intranodal	GCB	4	*BCL2*$^{GA/AMP}$	p.Y126_P128del	R-CHOP	PD	3.5
Case 154	59	Female	Intranodal	non-GCB	1	*BCL2*$^{GA/AMP}$	p.Tyr234His	R-CHOP-like	PR	7.5

GCB, germinal center-B cell like; non-GCB, non-germinal center-B cell like; MUT, mutation; GA, gain; AMP, amplification; COO, cell of origin; IPI, International Prognostic Index; PR, partial response; CR, complete response; PD, disease progression; PFS, progression-free survival.
